# Angiotensin-converting enzyme overexpression in mouse neutrophils prevents Alzheimer’s-like cognitive decline

**DOI:** 10.3389/fimmu.2026.1674330

**Published:** 2026-04-20

**Authors:** Tomohiro Shibata, Shabir A. Bhat, DuoYao Cao, Suguru Saito, Ellen A. Bernstein, Derick Okwan-Duodu, Warren G. Tourtellotte, Kenneth E. Bernstein, Zakir Khan

**Affiliations:** 1 Department of Pathology and Laboratory Medicine, Cedars-Sinai Medical Center, Los Angeles, CA, United States; 2 Department of Pharmacology, Yokohama City University School of Medicine, Yokohama, Japan; 3 Department of Pathology and Laboratory Medicine, David Geffen School of Medicine, University of California Los Angeles (UCLA), Los Angeles, CA, United States; 4 Department of Biomedical Sciences, Cedars-Sinai Medical Center, Los Angeles, CA, United States; 5 Department of Pathology, Faculty of Medicine, Stanford University, San Jose, CA, United States; 6 Department of Neurology, Cedars-Sinai Medical Center, Los Angeles, CA, United States; 7 Board of Governors Regenerative Medicine Institute, Cedars-Sinai Medical Center, Los Angeles, CA, United States; 8 Institute for Myeloma and Bone Cancer Research (IMBCR), West Hollywood, CA, United States

**Keywords:** Alzheimer’s disease, amyloid-beta, angiotensin converting enzyme (ACE), immune response, neutrophils

## Abstract

Angiotensin-converting enzyme (ACE), a dipeptidyl carboxypeptidase, is known to cleave amyloid-beta (Aβ), and its reduced activity has been linked to the progression of Alzheimer’s disease (AD). Our research indicates that ACE is vital for myeloid cell functions. Using ACE10/10 recombinant mice, we demonstrated that overexpressing ACE in macrophages mitigates AD pathology in these mice. Given that neutrophils are the most abundant white blood cells, this study investigates whether ACE overexpression in neutrophils influences AD progression. We crossed NeuACE mice, which overexpress ACE in neutrophils, with 3xTg-AD mice to create AD-NeuACE mice. Behavioral changes and brain pathology were assessed through various behavioral mazes and histological assays. AD-NeuACE mice demonstrated improved cognitive functions and lower Aβ levels in the cortex and hippocampus compared to AD mice. *In-vitro* data indicate that ACE-overexpressing neutrophils are significantly more effective at phagocytosing and clearing Aβ fluorescence particles. This study suggests that overexpressing ACE in neutrophils could be a promising approach to managing AD-like phenotypes.

## Introduction

Alzheimer’s disease (AD) is a relentlessly progressive and often fatal neurodegenerative disease that is the most common cause of dementia worldwide. While there is agreement that brain β-amyloid (Aβ) accumulation and plaque formation are neurotoxic and promote AD, the precise sequence of events leading to disease is still not understood. At present, there is no effective treatment for AD, but treatment approaches have suggested reducing brain Aβ burden as a means of treating disease (1–3).

The peptidase angiotensin-converting enzyme (ACE) is best known for regulating blood pressure as part of the renin-angiotensin system (RAS), in which ACE converts angiotensin I into the vasoconstrictor angiotensin II (4). ACE has many other effects, including the catalytic conversion of neurotoxic Aβ_1–42_ to the less toxic Aβ_1-40_ (5). Studies in humans suggest that blocking the RAS, and in particular blocking the effects of angiotensin II, reduces the incidence of AD. Surprisingly, blockers of the major angiotensin II receptor, the AT1 receptor, appear to be more effective in preventing AD than inhibitors of ACE (6–11). In fact, the role of ACE in AD is complicated by the finding that in a mouse model termed ACE 10/10, *increased* ACE expression by monocytes and macrophages is associated with significantly less disease when placed onto a mouse AD background as compared to AD mice having wild-type (WT) ACE levels (12, 13).

In general, brain inflammation, including that induced by AD, elicits peripheral immune cells, including monocytes, T cells, and neutrophils, to cross the blood–brain barrier (BBB) into brain parenchyma, where they may contribute to inflammatory damage and cognitive decline (14, 15). Concerning neutrophils and AD, studies in mice indicate that neutrophils can be found associated with Aβ plaques in the brain and that this may be deleterious since reduction of neutrophil numbers using anti-neutrophil antibody appeared to increase spatial short-term memory (16–19). In humans, neutrophils were observed associated with or near Aβ plaques and also near sites of BBB dysfunction (18, 20). These findings suggest that neutrophils may contribute to neuroinflammation and BBB breakdown associated with cognitive decline in AD patients (14). Neutrophils from 3xTg AD mice display defective phagocytosis of Aβ plaques and increased pro-inflammatory cytokine release (21). However, there is very little known about Aβ phagocytosis and clearance by neutrophils, and neutrophil function in AD remains significantly understudied.

We previously investigated the function of ACE in neutrophil function using a genetically engineered mouse line, known as NeuACE mice, in which neutrophils express increased ACE. In these animals, the ACE levels in monocytes and macrophages are comparable to those in WT mice (22, 23) (**Supplementary Figure 2D**). NeuACE mice have an enhanced immune response characterized by significantly reduced disease after challenge with bacterial infections, which is directly attributable to enhanced neutrophil function (22, 23). Neutrophils and macrophages differentiate from a common myeloid progenitor, and these cells have many similarities. ACE overexpression in either cell type induces a better immune response, at least as measured by bacterial challenge. Here, we studied NeuACE mice crossed with the transgenic 3xTg-AD mouse model of AD (AD^+^) to explore whether ACE overexpression in NeuACE neutrophils reduces AD pathology.

## Methods

### Mice

NeuACE mice were previously described (23). NeuACE transgenic mice were generated on a C57BL/6 background by inserting the mouse c-fms promoter upstream of mouse ACE cDNA. Among several founder lines screened, the NeuACE line demonstrated exclusive ACE overexpression in neutrophils without off-target expression in other tissues. The ACE genotypes studied are WT (WT/WT), heterozygous (NeuACE/WT), and homozygous for the targeted allele (NeuACE/NeuACE). These mice were backcrossed at least ten generations to C57BL/6J mice (The Jackson Laboratory). The 3xTg-AD strain was originally purchased from the Jackson Laboratory [B6;129-Tg (APPSwe, tauP301L)1Lfa Psen1tm1Mpm/Mmjax], then bred and maintained at Cedars-Sinai Medical Center. These mice are homozygous for the Psen1 mutation and also homozygous for the co-injected APPSwe and tauP301L transgenes with tissue expression restricted to the central nervous system. 3xTg-AD (AD^+^; C57BL/6J background) mice were crossed with NeuACE mice to produce the following genotypes: AD^+^ACE: WT/WT, AD^+^ACE: NeuACE/WT, AD^+^ACE: NeuACE/NeuACE. Male mice for each genotype were assessed at about 10 months old. All mice were maintained in microisolator cages, and all experiments were conducted and recorded by researchers blinded to the mouse genotypes.

### Flow cytometry analysis

For flow cytometry (FCM) analysis, blood neutrophils were purified with EasySep™ Mouse Neutrophil Enrichment Kit (19762, STEMCELL) following the manufacturer’s protocol. Cells were stained with APC/Fire750-anti-CD11b antibody (101262, BioLegend, 1:100) and PE/Cy7-anti-Ly6G antibody (108423, BioLegend, 1:100). For ACE immunostaining, we first stained cells with goat anti-ACE antibody (R&D Systems, AF1513, 1:100) as a primary antibody, followed by Alexa Fluor 647 anti-goat secondary antibody (Invitrogen, 21447, 1:500). Brains were perfused *in situ* with cold phosphate buffered saline (PBS), dissected, and mechanically dissociated in HBSS using a gentleMACS dissociator, followed by enzymatic digestion with collagenase D (1 mg/ml) and DNase I (50 μg/ml) for 30 min at 37 °C with gentle rotation. The resulting cell suspension was passed through a 70-μm cell strainer, and myelin was removed by 30% Percoll gradient centrifugation. The leukocyte-enriched fraction was collected from the interphase, washed, and resuspended in FACS buffer (PBS, 2% FBS, 2 mM EDTA). To exclude dead cells, samples were incubated with a fixable viability dye according to the manufacturer’s instructions. For surface staining, cells were blocked with anti-CD16/32 (Fc block) for 10 min at 4 °C and then stained for 30 min at 4 °C with fluorochrome-conjugated antibodies against CD45, CD11b, Ly6G (for neutrophils), CXCR2, CXCR4, CD80, CD86, and MHC class II. Brain-infiltrating neutrophils were identified as live CD45+ CD11b+, and expression of CXCR2, CXCR4, CD80, CD86, and MHC class II was quantified as median fluorescence intensity (MFI) and percentage of positive cells within the neutrophil gate. Following primary and secondary antibody immunostaining protocols, cells were resuspended in FCM buffer and analyzed immediately on the CYTEK NL-3000. Data was analyzed with FlowJo software. FCM data are presented as mean fluorescence intensity (MFI) (24). We presented data as fold change of MFI (relative to AD), in which individual data points were divided by the average of the AD values.

### Barnes maze test

We performed the Barnes maze test as described previously (13, 25). The mice were first trained for 4 days (acquisition-training phase), 2 times per day for 4 min for each trial per mouse. Following a 2-day break, the mice were retested for memory retention (retention phase, Day 7) and then short-term memory (reversal phase on Days 8 and 9) under the same conditions. The total latency was the duration of time between removal of the cylinder and animal entry into the escape tunnel. The primary latency was the time to locate the escape tunnel. The primary errors and total errors were the number of incorrect holes checked prior to locating (primary errors) and entering (total errors) the escape tunnel. Latency times and incorrect entries (errors) were recorded manually and by using a video camera.

### T-maze test

T-maze tests were performed according to the established methods (26, 27). Here, each arm of the maze was labeled as either A, B, or C. In each session, the animal is placed in arm A and allowed to explore the three arms for 5 min. We tested 2 times for 5 min per mouse. Activity in the T-maze was recorded by a video camera. Numbers of arm entries are scored from the recorded video file to calculate the percent alternation. The alternation percentage is calculated by dividing the number of alternations by the number of possible triads × 100. The maze was cleaned with Virkon solution between animals to eliminate odor traces.

### IHC staining of Ab

Paraffin-embedded tissue blocks were prepared by the immunohistochemistry lab at Cedars-Sinai Medical Center. The histology sections (5 μm) were heated for antigen retrieval in 10 mM sodium citrate (pH 6.0). Sections were subsequently exposed to specific mouse mAβ antibody for human Aβ (Sigma-Aldrich, A8354; 1:100). The slides were exposed to HRP-anti-mouse IgG (Sigma-Aldrich, A0168; 1:500) and then developed using the DAB Substrate Kit (VECTASTAIN, SK-4100). All slides were subsequently counterstained with Mayer’s hematoxylin. Determination of Aβ staining areas was assessed by ImageJ software.

### Immunofluorescence staining

Brain sections (5 μm thickness) from NeuACE-3xTg-AD were deparaffinized, rehydrated, and subjected to antigen retrieval using citrate buffer (pH 6.0) at 95 °C for 20 min. Sections were blocked with 5% normal goat serum in PBS containing 1% Triton X-100 for 20 mins at room temperature, then incubated overnight at 4 °C with primary antibodies: anti-β-amyloid (1:200, Moab-2, NBP2-13075, Novus Biologicals), anti-Ly6G (1:100, clone 1A8, BE0075-1, BioXCell), and anti-ACE/CD143 (1:150, orb216086, Biorbyt). Following washes in PBS, sections were incubated with species-appropriate Alexa Fluor-conjugated secondary antibodies (1:500, Thermo Fisher Scientific) for 1h at room temperature. Nuclei were counterstained with DAPI (1:1000). Slides were mounted with ProLong Gold antifade reagent (Invitrogen) and imaged using a Zeiss LSM 700 confocal microscope. Images were analyzed with Zen software for colocalization.

### Phagocytosis assay

Blood neutrophils were isolated using the EasySep Mouse Neutrophil Enrichment Kit (STEMCELL Technologies, Cat.19762). These neutrophils (1.0 × 10^5^ cells) were then placed in a 24-well plate and incubated with FAM-labeled Aβ1-42 (AS-23526-01, AnaSpec) for 30 min. Cells were then washed with FCM buffer and either analyzed immediately (0h) or incubated for an additional 2h. The analysis of the neutrophils was conducted using a flow cytometer and a fluorescent microscope.

## Results

### ACE overexpressed neutrophils prevent AD cognitive decline

**Figure 1:**
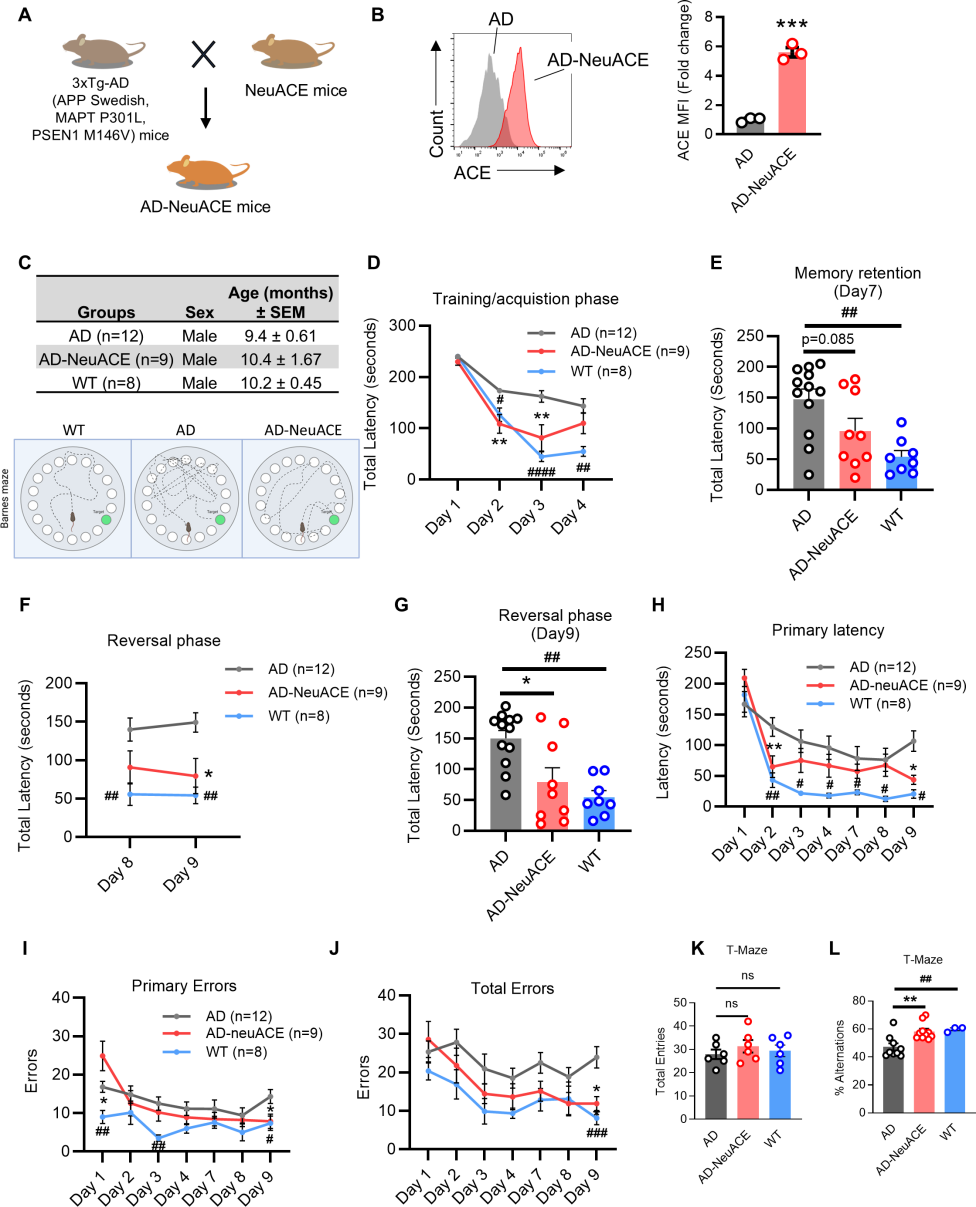
Analysis of cognitive function in AD-NeuACE and AD mice. **(A)** The creation of AD-NeuACE mice was achieved by crossing NeuACE mice with 3xTg-AD mice. **(B)** ACE expression in neutrophils from AD and AD-NeuACE mice was examined using flow cytometry. The results are displayed as a histogram (left) and mean fluorescence intensity (MFI) (right). Data analysis was performed using a two-sided unpaired Student’s t-test, with data represented as means ± SEM (*n* = 3, ***p < 0.001). **(C)** The quantity of mice per genotype and age group (AD, AD-NeuACE, and WT) used in the behavior experiment is detailed. **(D–G)** Cognitive function in AD, AD-NeuACE, and WT mice (5–12 mice/group) was evaluated using the Barnes maze test. The data is represented as total latency (seconds) during the acquisition/training phase (Days 1–4) **(D)**, memory retention (Day 7) **(E)**, reversal phase (Days 8–9) **(F)**, and memory test on Day 9 **(G)**. **(H–J)** The Barnes maze test was also utilized to assess cognitive function in AD, AD-NeuACE, and WT mice (5–12 mice/group). The data is represented as primary latency (seconds) **(H)**, total incorrect entries (errors) **(I)**, and primary errors **(J)** during the acquisition/training phase (Days 1–4), memory retention (Day 7), and reversal phase (Days 8–9). **(K, L)** The total number of arm entries and the total spontaneous alternations over a 5-min period in the T-maze test are displayed. Each dot signifies an individual animal. **(D–K)** Group comparisons were analyzed using a one-way ANOVA with Bonferroni’s correction for multiple comparisons, with data represented as means ± SEM. * or ^#^*p* < 0.05, ** or ^##^*p* < 0.01, *** or ^###^*p* < 0.001, (*AD vs. NeuAD; ^#^ AD vs. WT).

To study AD in NeuACE mice, these animals were crossed with 3xTg-AD mice. This is a well-established mouse model for AD, in which animals overproduce human Aβ peptides, develop plaques with age, and show progressive memory and learning deficits mimicking aspects of human AD. Mouse breeding eventually yielded 3xTg-AD (AD^+^) heterozygous mice that were homozygous for WT ACE (termed AD) and AD^+^ heterozygous mice that were homozygous for the NeuACE allele (termed AD-NeuACE) (**Figure 1A**). We did not study mice heterozygous for the NeuACE allele.

We first assessed ACE expression in AD-NeuACE neutrophils, which was approximately sixfold higher than ACE levels in AD neutrophils, as determined by FCM analysis (**Figure 1B**; **Supplementary Figure 1**). Blood pressure measurements showed no differences between NeuACE and control mice, confirming that neutrophil-specific ACE overexpression does not perturb cardiovascular homeostasis. Next, we assessed the cognitive function of AD-NeuACE mice as compared to AD and WT (no AD) mice using a Barnes maze, which tests the hippocampal-based ability to learn and remember the location of an escape tunnel using spatial cues (13). Mice were gender-matched and were assessed at 9.4 to 10.4 months of age (**Figure 1C**). During the training/acquisition phase of 4 days, AD mice showed impaired training compared to WT mice, while AD-NeuACE mice displayed a pattern of maze learning that was intermediate to that of AD and WT mice (**Figure 1D**). On Day 7, analysis of memory retention showed a similar pattern: there was a substantial difference in average total latency between AD and WT mice (**Figure 1E**, *p* < 0.01). While the average total latency of the AD-NeuACE mice was intermediate to the two other groups, the difference between AD and AD-NeuACE mice was not significant (*p* = 0.085). Following the reversal phase, memory retention analysis on Day 9 found a significant difference between AD and AD-NeuACE mice, with AD-NeuACE mice demonstrating improved cognitive behavior as indicated by reduced total latency (**Figures 1F, G**). We also assessed the primary latency, primary errors, and total errors in the Barnes maze tests and found that AD-NeuACE mice displayed a pattern of learning significantly better than AD mice (**Figures 1H–J**). Spatial function and short-term memory were also evaluated using a T-maze spontaneous alternation test (27), which assesses the number of times a mouse explores alternative arms of the maze. There were no significant differences in the total number of arm entries among the three groups, indicating comparable levels of general activity and exploration (**Figure 1K**). However, AD-NeuACE mice displayed a significantly higher percentage of spontaneous alternations compared with AD mice, approaching levels observed in WT animals (**Figure 1L**). These results suggest that neutrophil-specific overexpression of ACE improves short-term memory and spatial cognition in the 3xTg-AD model. Together with findings from the Barnes maze, these data support the conclusion that enhanced ACE activity in neutrophils confers cognitive benefits and may attenuate the progression of AD-like symptoms.

### Reduced Aβ plaque in NeuACE mice

**Figure 2:**
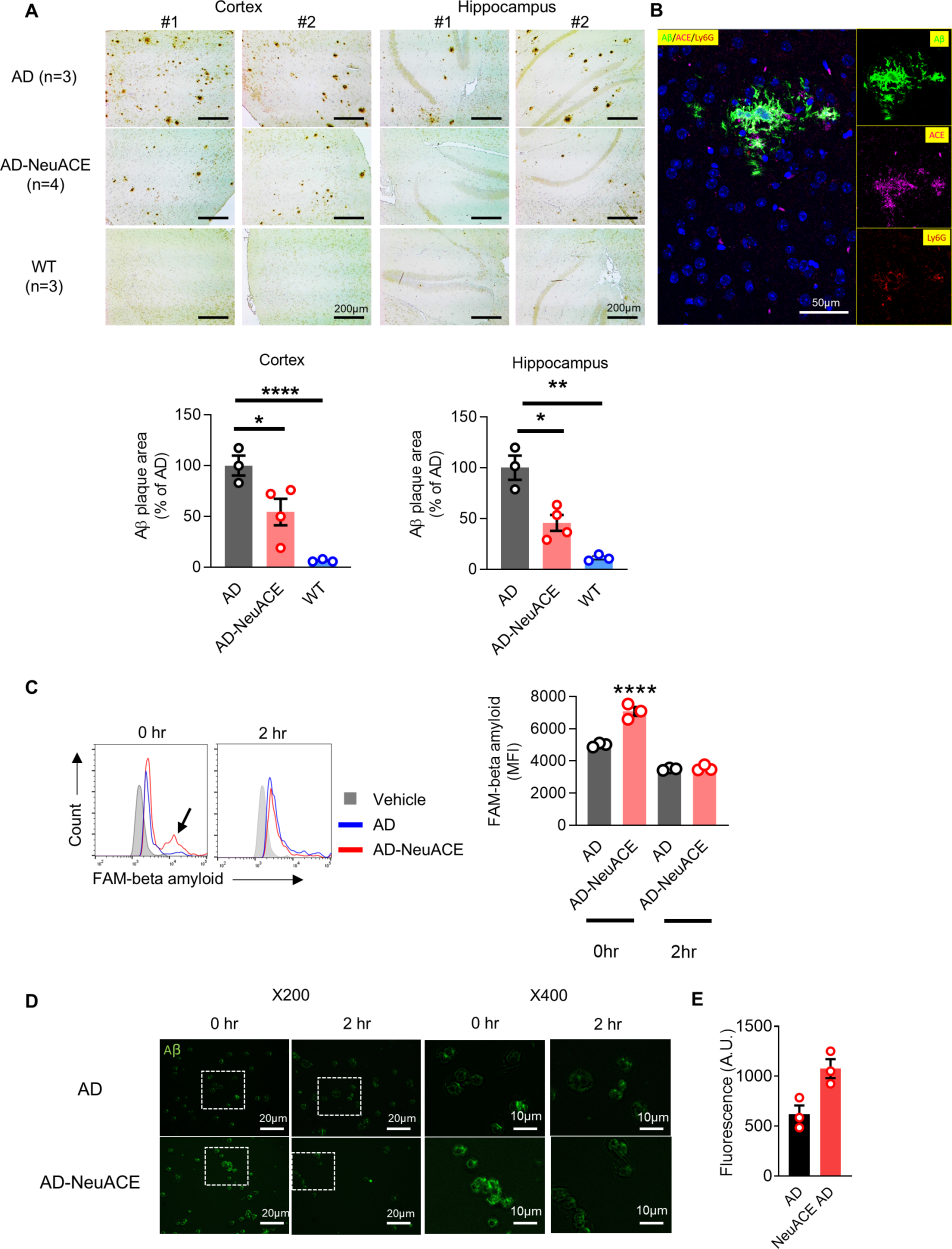
Overexpression of ACE in neutrophils correlates with decreased Aβ levels. **(A)** Histological examination of Aβ in the cortex and hippocampus of AD, AD-NeuACE, and WT brains was conducted. The magnification used was ×100, and the scale bar represents 200 µm. The intensity of Aβ staining was analyzed using ImageJ software, and the quantitative data from all samples are displayed in the bar chart. Each dot on the graph signifies an individual sample. Group comparisons were analyzed using a one-way ANOVA with Bonferroni’s correction for multiple comparisons, and data are represented as means ± SEM. *p < 0.05, **p < 0.01, and ****p < 0.0001. **(B)** Representative immunofluorescence images showing colocalization of ACE1 (magenta), Ly6G+ neutrophils (red), and Aβ plaques (green) in hippocampal sections. **(C, D)** *In-vitro* phagocytic degradation analysis of fluorescent (FAM)-labeled β-Amyloid (FAM-Aβ). Blood neutrophils were incubated with FAM-Aβ for 30 min. After washing the neutrophils with FCM buffer, the FAM intensity of the neutrophils was assessed immediately (0h) or after a 2h incubation using flow cytometry **(B)** or by fluorescent microscope **(C)**. Comparisons were analyzed using a two-sided unpaired Student’s t-test, and data are represented as means ± SEM (*n* = 3). **p < 0.01.

The accumulation of Aβ is the key step that accelerates neurodegeneration in AD (2, 28). To examine this, mice were sacrificed at 10 months of age for brain immunohistochemistry using a mouse anti-human Aβ antibody to quantify plaque burden. Quantitative image analysis of cortical and hippocampal Aβ plaque areas revealed significantly decreased plaque areas in AD-NeuACE mice as compared to AD mice (**Figure 2A**, *p* < 0.05).

To explore the cellular basis of this reduction and get a clue from the previous findings, where macrophages overexpressing ACE (ACE 10/10 cells) phagocytize and eliminate Aβ more effectively than WT cells, we analyzed the Aβ-clearing capacity of neutrophils isolated from AD and AD-NeuACE mice. Immunofluorescence staining of NeuACE-3xTg-AD brain sections revealed ACE1+ neutrophils (Ly6G+) localized adjacent to Aβ plaques, confirming neutrophil-specific ACE1 expression in proximity to pathology (**Figure 2B**). Bone marrow–derived AD and AD-NeuACE neutrophils were incubated *in vitro* with FAM-Aβ for 30 min to measure uptake of Aβ as determined by FCM (**Figure 2C**, time 0). This showed increased uptake by a subset of AD-NeuACE neutrophils. Cells were also studied for FAM-Aβ after an additional 2h to measure Aβ elimination. The increased Aβ signal observed in AD-NeuACE cells was markedly reduced, indicating more efficient degradation of internalized Aβ. These findings were further supported by fluorescence microscopy, which confirmed a greater degree of Aβ uptake and clearance in AD-NeuACE neutrophils compared to controls (**Figure 2D**). This confirmed the FCM data, particularly how effective the Neu-ACE cells are in degrading Aβ.

**Figure 3:**
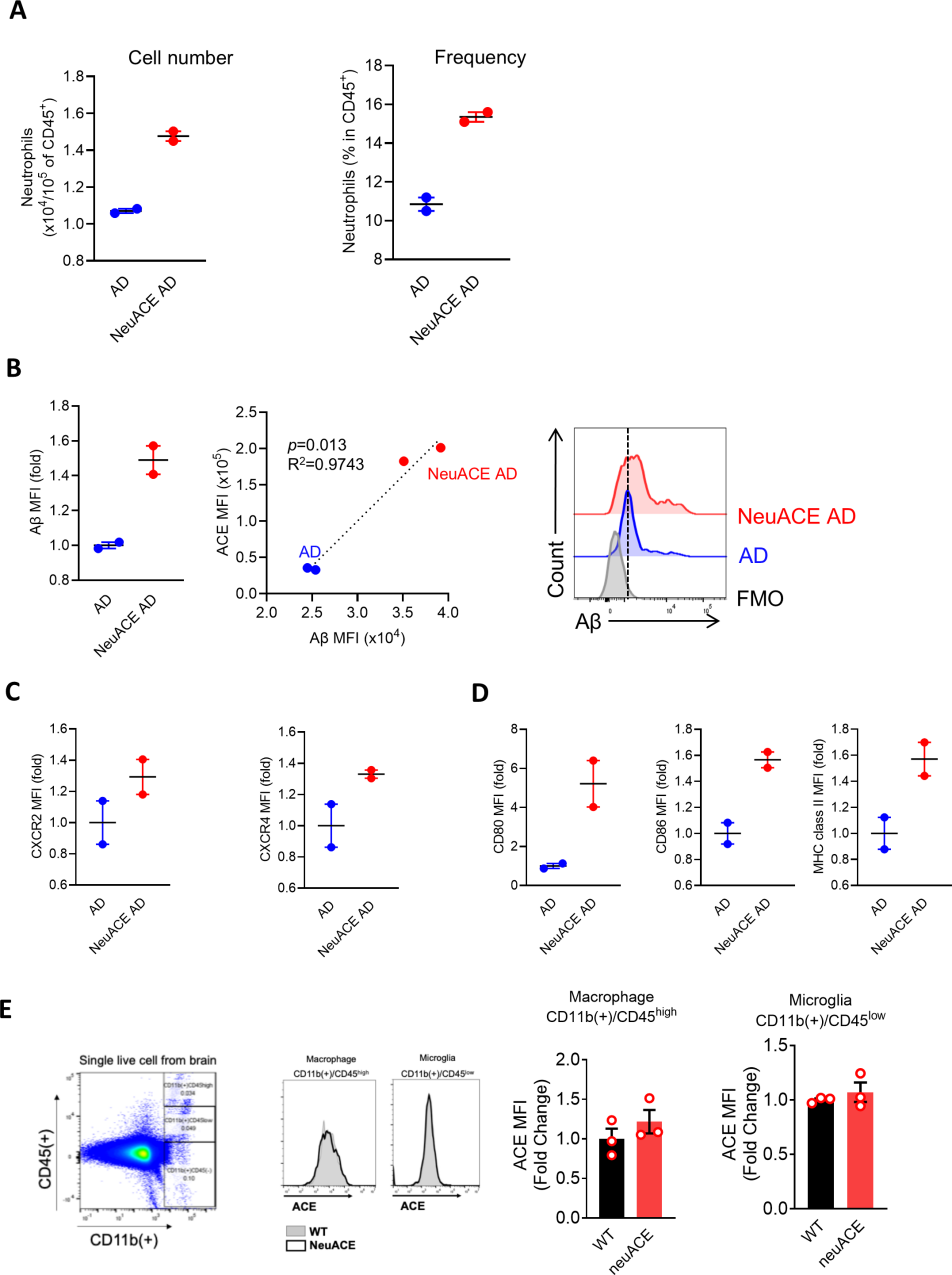
Flow cytometry analysis of brain-infiltrating neutrophils and resident myeloid cells in AD and AD-NeuACE mice. **(A)** Quantification of neutrophil infiltration showing (left) absolute cell numbers (×104 CD45+) and (right) frequency as percentage of total CD45+ cells. **(B)** Amyloid-β uptake by brain neutrophils displayed as (left) fold change in Aβ mean fluorescence intensity (MFI), (middle) correlation between Aβ MFI and neutrophil frequency, and (right) representative flow cytometry histograms showing Aβ signal in AD (blue) versus AD-NeuACE (red) neutrophils compared to fluorescence-minus-one (FMO) control. **(C)** Expression of chemokine receptors CXCR2 (left) and CXCR4 (right) shown as MFI fold change. **(D)** Activation markers on brain neutrophils: CD80 MFI (left), CD86 MFI (middle), and MHC class II MFI (right). **(E)** ACE expression in brain myeloid cells: (left) representative flow cytometry plots showing gating strategy for macrophages (CD11b+CD45high) and microglia (CD11b+CD45low), (middle) ACE MFI in brain macrophages, and (right) ACE MFI in microglia, demonstrating no change in ACE expression in these populations in NeuACE mice. All data shown as mean ± SEM (n=2/3 per group). Statistical comparisons performed using two-sided unpaired Student's t-test.

FCM analysis revealed distinct functional phenotypes in neutrophils isolated from NeuACE AD mice compared to conventional AD mice. NeuACE AD mice demonstrated significantly enhanced neutrophil amyloid-beta (Aβ) uptake within the brain parenchyma (**Figures 3A, B**). This increased phagocytic capacity was accompanied by elevated expression of chemokine receptors CXCR2 and CXCR4, indicating enhanced migratory potential toward sites of pathology (**Figure 3C**).

Furthermore, neutrophils from NeuACE AD mice exhibited markers of increased activation, including upregulation of costimulatory molecules CD80 and CD86, as well as elevated major histocompatibility complex class II (MHC-II) expression (**Figure 3D**). These findings suggest that neutrophils in the NeuACE model adopt a more activated, antigen-presenting phenotype compared to those in standard AD mice (**Figure 3**).

## Discussion

A large number of clinical studies have suggested that systemic inflammation is associated with greater cognitive decline in AD (29). Neutrophil accumulation has also been associated with cognitive decline in both mice and humans (14). Increased neutrophil extracellular traps (NETs) released by neutrophils are observed in AD and are considered one of the possible mechanisms for neutrophil BBB breaching and neuronal damage (15, 18, 30). Further, reduced neutrophil phagocytosis has been observed in late-stage AD patients with cognitive impairment (31, 32). Neutrophils in the brains of AD mice were observed near or at Aβ plaques and were found near sites of BBB dysfunction (29).

In the present study, we found that ACE overexpression in AD-NeuACE mice promotes phagocytosis of Aβ by neutrophils and reduces cognitive decline as compared to AD mice. Increased ACE appears to convert neutrophils from a potential cause of neural injury into cells capable of protecting against AD pathology, and thus, some form of ACE-overexpressing neutrophils may be envisioned as a potential AD therapy. At the very least, this study complements previous analysis, which showed that macrophages expressing increased ACE were also highly efficacious in reducing pathology in a mouse model of AD (13). In this model, elevated myeloid cell function seems to consistently reduce AD injury, including cognitive decline.

In terms of the mechanism for how ACE expression affects AD pathology, it has been observed that ACE can break down Aβ (5). Furthermore, a previous study has shown that proteins in neutrophil granules can bind to Aβ and prevent it from aggregating (33). How ACE interacts with these proteins is a crucial area of future study. Our research has also revealed that ACE boosts the antibacterial activity of human neutrophils (22, 23). Our findings align with recent work demonstrating that ACE overexpression in microglia enhances Aβ clearance and improves cognitive outcomes in AD models (34). Together, these studies suggest that ACE acts as a potent enhancer of myeloid cell function across distinct lineages, including both brain-resident microglia and circulating neutrophils. The relatively small sample size may limit the generalizability of our findings and warrants cautious interpretation. Future studies with larger cohorts are needed to confirm and extend these results. Therefore, our findings underscore the physiological significance of ACE in neutrophil function. They also suggest that enhancing the function of neutrophils and macrophages could be an effective strategy for reducing AD pathology.

Future investigations should assess whether neutrophil-mediated amyloid reduction influences tau pathology in the 3xTg-AD model. Analysis of tau phosphorylation markers at later time points would clarify whether enhanced amyloid clearance provides comprehensive disease-modifying effects or whether combination strategies targeting both amyloid and tau pathways are required for optimal therapeutic outcomes.

## Data Availability

The raw data supporting the conclusions of this article will be made available by the authors, without undue reservation.

## Supplementary material

Supplementary Figure 1 **(A)** Gating strategy for the neutrophil analysis for **Figure 2A**. **(B)** Gating strategy for the neutrophil analysis for **Figure 2B**.
