# Supplementary figures and images for: Angiotensin-converting enzyme overexpression in mouse neutrophils prevents Alzheimer’s-like cognitive decline
Source: Front Immunol. 2026 Apr 20;17:1674330. doi: 10.3389/fimmu.2026.1674330 (PMC13138015; doi:10.3389/fimmu.2026.1674330)

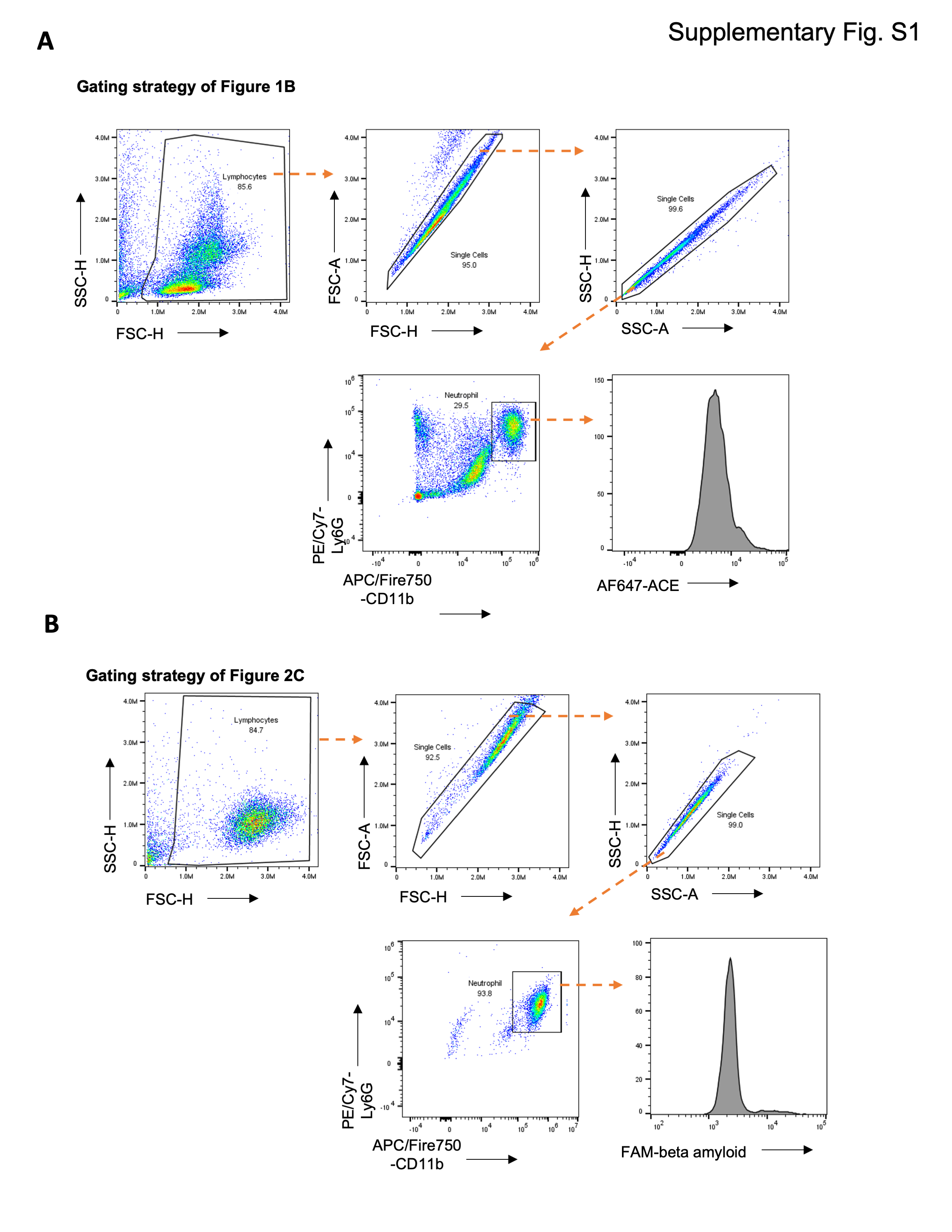

Supplement: Supplementary Figure 1 — (A) Gating strategy for the neutrophil analysis for Figure 2A. (B) Gating strategy for the neutrophil analysis for Figure 2B. [file Image1.tiff]
